# Diverse Mechanisms Underlie Enhancement of Enteric Viruses by the Mammalian Intestinal Microbiota

**DOI:** 10.3390/v11080760

**Published:** 2019-08-17

**Authors:** Alexa N. Roth, Katrina R. Grau, Stephanie M. Karst

**Affiliations:** Department of Molecular Genetics & Microbiology, Emerging Pathogens Institute, Center for Inflammation and Mucosal Immunology, College of Medicine, University of Florida, Gainesville, FL 32610, USA

**Keywords:** intestinal microbiota, enteric virus, virus–bacteria interaction

## Abstract

Over the past two decades, there has been tremendous progress in understanding the impact of the intestinal microbiota on mammalian metabolism, physiology, and immune development and function. There has also been substantial advancement in elucidating the interplay between commensal and pathogenic bacteria. Relatively more recently, researchers have begun to investigate the effect of the intestinal microbiota on viral pathogenesis. Indeed, a growing body of literature has reported that commensal bacteria within the mammalian intestinal tract enhance enteric virus infections through a variety of mechanisms. Commensal bacteria or bacterial glycans can increase the stability of enteric viruses, enhance virus binding to host receptors, modulate host immune responses in a proviral manner, expand the numbers of host cell targets, and facilitate viral recombination. In this review, we will summarize the current literature exploring these effects of the intestinal microbiota on enteric virus infections.

## 1. Introduction

Enteric viruses infect the gastrointestinal (GI) tract following fecal-oral transmission and are collectively responsible for a tremendous disease burden. They are often highly infectious and shed in significant quantities in the stool of infected individuals. Rotaviruses, noroviruses, and astroviruses are all nonenveloped RNA enteric viruses and causative agents of acute gastroenteritis [1,2]. Rotaviruses cause severe diarrhea in infants and children under five years of age worldwide [1,2]. Prior to the implementation of an effective vaccination program, they were responsible for an estimated 500,000 deaths globally per year, particularly in low-income countries [3,4]. This fatality rate has been markedly reduced due to vaccination, with a current estimated global death rate of 200,000 worldwide per year [5]. As such, noroviruses have emerged as the most common cause of severe childhood diarrhea [6,7], in addition to being the leading cause of foodborne disease and gastroenteritis outbreaks in all age groups [8]. Astroviruses are another major cause of pediatric gastroenteritis and are increasingly recognized as a leading cause of diarrhea in immunocompromised patients [9]. Certain picornaviruses also infect the GI tract, causing gastroenteritis and in some cases extraintestinal disease. For example, after replicating in the GI tract poliovirus can disseminate from the intestine to the central nervous system and cause paralytic poliomyelitis in a small proportion of infected individuals [2,10].

The mammalian gut lumen is colonized by a vast population of microorganisms, referred to collectively as the intestinal microbiota. It consists of trillions of microbes from more than 1000 different bacterial species in addition to viruses, fungi, and archaea [11,12]. The intestinal microbiota and their metabolites are well-established to play significant roles in host physiology and homeostasis, metabolism, and nutrient uptake [13,14,15,16,17]. Furthermore, they are critical to normal immune development and functionality [18,19], aiding in the development of the gut-associated lymphoid tissue (GALT) [14,20,21,22] and influencing the development and function of mucosal T cells [23]. Finally, they can provide protection from pathogenic bacteria through a phenomenon coined colonization resistance, in which commensal bacteria compete with pathogenic ones for nutritional niches, produce inhibitors that target pathogens, and induce broad-spectrum antimicrobial immune responses.

Considering that all enteric viruses encounter the intestinal microbiota as they traverse the GI tract, it is imperative to consider how virus–bacteria interactions may impact the outcome of viral infections. Indeed, two landmark studies published in *Science* in 2011 demonstrated that commensal bacteria stimulated poliovirus, reovirus, and mouse mammary tumor virus (MMTV) infections in the intestines of mice [24,25]: Mice treated with oral antibiotics prior to poliovirus infection had reduced viral shedding, decreased virus titers, and reduced disease severity compared to conventionally colonized mice, indicating that the intestinal microbiota facilitated poliovirus pathogenesis [25]. Similarly, reovirus replicated less efficiently and caused reduced intestinal and liver disease in antibiotic-treated mice compared to control mice [25]. MMTV is a retrovirus transmitted via fluids and often ingested by mouse pups through milk from chronically infected mothers, leading to the establishment of initial infection in the gut [26]. Unlike conventional mice, germ-free MMTV-infected dams were unable to transmit virus to their offspring, demonstrating a critical role for commensal bacteria in viral transmission [24]. Similar findings have been reported for noroviruses and rotavirus [27,28,29,30]: An intact microbiota contributed to increased acute murine norovirus (MNV) infection in the distal small intestine [27] and promoted the establishment of persistent MNV infection in the colon [28,29]. Furthermore, human norovirus replication in cultured B cells is enhanced by commensal bacteria [27]. Finally, rotavirus disease severity and infectivity were reduced in antibiotic-treated and germ-free mice compared to conventionally colonized mice [30]. Overall, the intestinal microbiota enhanced the pathogenesis of multiple families of enteric viruses. Recent studies revealed that these viruses have evolved unique and varied strategies to exploit commensal microbes and enhance their efficiency at infecting mammalian hosts.

## 2. Mechanisms of Bacterial Enhancement of Enteric Virus Infections

**Figure 1:**
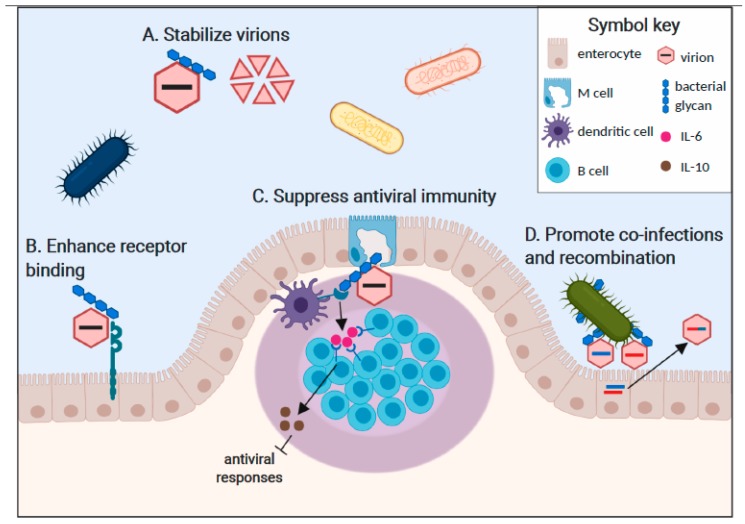
Commensal bacteria enhance enteric virus infections in multiple ways. **A.** Virion binding to bacterial glycans increases particle stability in the face of environmental stresses and thereby enhances host-to-host transmission efficiency. Poliovirus, reovirus, and norovirus particles are stabilized by bacterial ligands. **B.** Direct interactions of glycan-bound viral particles with cellular entry receptors enhance the stability of capsid-receptor interactions and promote initiation of infection. A specific interaction between poliovirus, its receptor (PVR), and bacterial LPS has been reported. **C.** Immune sensing of commensal bacterial components results in a tolerogenic gastrointestinal microenvironment that promotes enteric virus replication. For example, dendritic cell and macrophage sensing of LPS-bound MMTV through TLR4 results in the release of IL-6, which stimulates B cells to express the anti-inflammatory cytokine IL-10. **D.** Multi-virion clustering on bacterial surfaces increases the frequency of viral co-infection of a single cell, driving recombination potential that can result in enhanced fitness of progeny recombinant virus strains.

Although the mechanisms underlying commensal bacterial regulation of viral infections are less well-defined than for bacterial pathogens, a multitude of varied mechanisms have begun to emerge. While it is unclear whether all of these mechanisms require direct interaction between the enteric virus and commensal bacteria, certain mammalian enteric viruses including poliovirus [31], reovirus [32], and norovirus [33,34] have been visualized attached to the surface of bacteria. Poliovirus and MMTV directly bind to bacterial lipopolysaccharide (LPS) [24,25] which is present on the outer membrane of Gram-negative bacteria and can be shed from the bacterial surface as a free molecule in the GI lumen. In terms of the mechanism of attachment, MMTV incorporates LPS binding proteins including CD14, TLR4, and MD-2 into its viral envelope as it buds from host cells [35] while nonenveloped poliovirus capsids bind LPS directly [25]. Human norovirus binds to histo-blood group antigens (HBGA), neutral glycans expressed on the surface of many commensal microbes [34]. These viruses are well-established to bind HBGA at a surface-exposed domain of their VP1 capsid protein and can also bind host-derived HBGA [36]. Another study demonstrated human norovirus binding to HBGA-negative bacteria, raising the possibility of additional attachment factors or virus strain differences in attachment factor usage [33]. Reovirus can associate with Gram-positive and Gram-negative bacteria although the precise ligand is unknown [32]. In this review, we will provide a detailed summary of our current understanding of specific mechanisms of bacterial enhancement of mammalian enteric virus infections (Figure 1): First, bacterial glycans can stabilize virions [32,37]. Second, bacterial glycans can enhance virus attachment to target cells [27,32,37]. Third, bacterial interactions with enteric viruses can regulate antiviral immune responses in a proviral manner [24,28,35]. Fourth, bacterial interactions can facilitate viral co-infections of target cells and subsequent viral recombination [31].

### 2.1. Bacterial Stabilization of Virus Particles

One consequence of virion binding to bacterial glycans is particle stabilization (Figure 1A). For example, incubation of poliovirus particles with bacterial surface N-acetylglucosamine (GlcNAc)-containing polysaccharides longer than six units including LPS, peptidoglycan (PG), and chitin enhanced their thermostability and resistance to inactivation by diluted chlorine bleach [25,37]. This increased stability correlated with delayed release of viral genome from the particle, suggesting that bacterial stabilization prevents premature genome extrusion prior to virus binding to host cells [37]. LPS interaction with the poliovirus VP1 capsid protein is critical for particle integrity, as indicated by the reduced stabilization of virions containing a threonine-to-lysine mutation at residue 99 of VP1 (T99K virus). Reovirus also displayed enhanced thermostability in the presence of LPS, PG, mannan, and mucin; while lipoteichoic acid and chitin had moderate stabilizing effects on certain reovirus strains [32]. This was true for mature reovirus particles as well as infectious subviral particles (ISVP) which are formed by proteolysis in the intestinal lumen of infected hosts. Incubation with LPS or PG did not interfere with nor enhance reovirus binding to its receptor, junctional adhesion molecule-A (JAM-A), nor did it prevent neutralization by virus-specific antibodies, suggesting that bacterial polysaccharides bind to a site on the virion distinct from the receptor binding site and neutralizing epitopes. For human noroviruses, the association of virus-like particles (VLPs) with HBGA-expressing *E. coli* resulted in improved capsid antigen integrity and mucin binding ability after heat treatment compared to VLPs alone or after incubation with a non-capsid binding *E. coli* strain [38]. Overall, bacterial polysaccharides confer enhanced stability to several mammalian enteric viruses. Considering that these viruses are shed in feces, maintained in the environment before being transmitted to another host, and then traverse the harsh conditions of the stomach and intestinal lumen before initiating infection of host intestinal cells, increased stability is likely to provide a substantial fitness advantage. Consistent with this, wild-type stabilization-competent poliovirus was transmitted more readily than T99K stabilization-incompetent virus in a co-infection setting [37].

### 2.2. Bacterial Enhancement of Virus Attachment to Target Cells

Another consequence of virus interactions with bacterial glycans is enhancement of virion binding to host cell receptors (Figure 1B). For example, the efficiency of poliovirus attachment to the surface of cells expressing the poliovirus receptor (PVR) and to soluble PVR were increased in the presence of LPS [37]. Further demonstrating the specificity for LPS in enhancing poliovirus binding to PVR, incubation of poliovirus with LPS failed to promote viral infection on non-PVR expressing cells; and blocking PVR with a specific antibody prevented poliovirus infection irrespective of the presence of LPS. Interestingly, the T99K mutation in the poliovirus VP1 capsid protein that reduced the LPS stabilizing activity had no effect on the ability of LPS to enhance PVR binding, demonstrating that the LPS stabilization of virions and LPS enhancement of poliovirus receptor binding are distinct mechanisms.

A similar model has been proposed for human norovirus infection of B cells [27]: HBGA-expressing commensal bacteria or recombinant HBGA, but not HBGA-negative bacteria or LPS, enhanced human norovirus infection of B cells. Moreover, recombinant HBGA were sufficient to facilitate human norovirus attachment to the surface of B cells. While these data are consistent with a model similar to LPS-mediated stimulation of poliovirus binding to PVR, the receptor(s) for human norovirus has not been defined so confirmatory studies are not yet possible. Human norovirus infection of B cells requires a commensal bacterial co-factor in the form of HBGA, while the virus can infect stem cell-derived enteroid cultures in the absence of bacteria and is instead dependent on host-derived HBGA [39]. Considering that both host epithelial cells and commensal bacteria express HBGA on their surfaces and secrete soluble HBGA into the gut lumen [40,41,42], it is fascinating to consider the complex interactions that impact human norovirus pathogenesis within a susceptible host.

Murine norovirus (MNV) attachment to its host receptor may be influenced indirectly by the intestinal microbiota. The MNV receptor has been identified as CD300lf [43,44], a molecule expressed on a variety of immune cells and tuft cells that are all targets of MNV *in vivo* [45,46]. Interestingly, MNV binding to CD300lf is enhanced by a bile acid cofactor [47]. Considering that commensal bacteria biotransform bile acids and regulate the bile acid pools within the intestinal lumen, it is tempting to speculate that bacterial metabolism modulates norovirus infection efficiency and may underlie, at least in part, the reduced ability of MNV to infect antibiotic-treated and germ-free mice [27,28,29]. Consistent with this concept, bile acids enhance infection of certain human norovirus strains in human intestinal enteroid cultures, murine norovirus in a microglial cell line, and the related porcine enteric calicivirus in immortalized porcine kidney cells [39,47,48].

### 2.3. Bacterial Modulation of Host Immunity in a Proviral Manner

Commensal bacteria are sensed by host intestinal epithelial and immune cells continuously, leading to the establishment of a tolerogenic microenvironment in the steady-state. Though the host immune system normally differentiates between commensal and pathogenic organisms to effectively mount an inflammatory response during infections, it is intriguing to consider what happens when a pathogen such as an enteric virus is attached to, and sensed concurrently with, a commensal bacterial ligand. One possibility is that the commensal bacterial signal will dampen or block the antiviral immune response via bystander suppression. Indeed, this appears to be the case during MMTV infections [24]: As described above, MMTV binds LPS through host LPS binding proteins (e.g., CD14, MD2, and TLR4) in its viral envelope that are acquired during the viral budding process. These LPS-bound MMTV virions, ingested in maternal milk by neonatal mice, stimulate TLR4 expressed on host cell surfaces, initiating a signaling cascade culminating in the production of anti-inflammatory IL-10. In a series of elegant studies, the Golovkina research group has shown that TLR4 engagement on dendritic cells and macrophages results in the production of IL-6, which then drives IL-10 expression by B cells [24,35,49]. Collectively, this results in a tolerogenic environment that allows viral persistence to be established in the pups (Figure 1C). Supporting the concept that LPS-bound MMTV induces tolerance to viral antigens, mice that became persistently MMTV-infected as pups failed to mount an antiviral antibody response when immunized with viral antigens as adults [24]. Bystander suppression may also occur during norovirus infections [50]: MNV infection of wild-type mice induced only modest inflammation which was significantly increased in infected IL10^−/−^ mice. Conversely, no inflammation was elicited in germ-free IL10^−/−^ mice infected with MNV though the inflammatory response could be rescued by colonizing the mice with a defined microbiota, demonstrating that MNV-induced inflammation in the absence of IL-10 is microbiota-dependent.

The intestinal microbiota also influenced the host interferon (IFN) response during persistent MNV infection in a manner promoting viral infection [28]: Whereas persistent MNV infection was reduced in antibiotic-treated wild-type mice and mice deficient in type I or type II IFN receptors, it was not affected by antibiotic treatment in mice lacking the type III IFN (IFN-λ) receptor; STAT-1 (a key IFN signaling molecule); or IRF3 (a transcription factor required for IFN gene expression). These findings suggest that commensal bacteria suppress IFN-λ responses, providing a favorable environment for viral persistence. A recent study by Wilen et al. suggests that the intestinal microbiota could play an additional stimulatory role in MNV persistence establishment [45]: Antibiotic treatment resulted in a reduction of colonic tuft cell numbers, a rare type of intestinal epithelial cell that serves as the persistent reservoir of MNV, suggesting that the intestinal microbiota could expand the numbers of host cell targets for a given enteric virus.

Likewise, the interplay of commensal microbes and host immunomodulatory functions intersected in a manner that influenced acute MNV replication. The disruption of secretory immunoglobulin (sIg) function in the gastrointestinal lumen led to a proinflammatory state of increased type II IFN (IFN-γ) and iNOS production and an altered microbial community in the gut compared to control mice [51]. MNV replication was reduced in specific regions of the intestine upon infection of dysbiotic polymeric immunoglobulin receptor (pIgR)^−/−^ mice [51]. The same was true for reovirus. Although the specific virus–bacteria interaction occurring in this system has yet to be identified, these data indicated a critical role for a steady-state commensal microbe community in enteric virus replication. It should be noted that acute and persistent MNV strains display strikingly distinct pathogenesis in spite of using the same virus receptor CD300lf, with acute MNV targeting a variety of intestinal immune cells in the gut-associated lymphoid tissue of the distal half of the small intestine [46] instead of colonic tuft cells [45]. It will be fascinating to elucidate whether acute and persistent MNV strains also differ in their interactions with the intestinal microbiota.

### 2.4. Bacterial Promotion of Viral Recombination

Efforts to visualize the binding of poliovirus, reovirus, and norovirus capsids to bacterial cells each noted the presence of multiple virions bound per bacterium [31,32,33,34]. In theory, this could effectively increase the multiplicity of infection (MOI) if virus-loaded bacteria contact a susceptible host cell. It could also provide an evolutionary advantage to viruses that exist as quasispecies by delivering multiple viral genomes per cell and increasing the possibility for genetic recombination [31,52]. Using genetically engineered poliovirus strains that expressed distinct fluorescent markers, Erickson et al. demonstrated that the incubation of a virus with bacteria indeed increased the frequency of viral coinfections [31]. Moreover, elegant studies using genetically distinct “barcoded” poliovirus strains proved that at least six genetically distinct polioviruses could infect one host cell under very low MOI conditions in the presence of bacteria; and that this coinfection enhanced viral recombination. Bacterially-enhanced coinfection of mammalian cells by a heat-resistant, drug-sensitive poliovirus strain in conjunction with a heat-sensitive, drug-resistant strain resulted in progeny virus that was both heat- and drug-resistant [31]. In summary, viral binding to bacteria can concentrate viral particles and facilitate coinfection of host cells, thereby increasing genetic recombination and virus adaptability (Figure 1D). Although this phenomenon has only been demonstrated experimentally for poliovirus, the observation of multi-virion binding to individual bacteria for other virus families [32,33,34] makes it tempting to speculate that this is a common mechanism used by enteric viruses to increase their effective MOI and enhance viral genetic diversity.

## 3. Discussion and Future Outlook

The intestinal microbiota is beneficial for the host in several aspects: Gut bacteria aid in host metabolism, gut motility, and nutrient uptake, maintain host physiology and homeostasis, help shape the host immune system, and protect against invading microorganisms. Although bacteria in the gut have been studied for decades, the majority of polymicrobial interaction studies have focused on pathogenic-commensal bacteria relationships, leaving the influence of commensal bacteria on enteric virus infection largely unexplored.

Recently, there has been a marked surge in reports exploring the effects of the host microbiota on enteric virus infection. These reports have a highlighted a stimulatory role for the commensal microbiota in the transmission and pathogenesis of numerous enteric viruses, including poliovirus, reovirus, rotavirus, MMTV, and norovirus. Although enhancement of viral infection by interactions with commensal bacteria has emerged as a phenomenon shared by multiple families of enteric viruses, there are myriad mechanisms of interaction through which enhancement occurs. Broadly these mechanisms include: The stabilization of viral particles by bacterial ligands aiding in host-to-host transmission; enhancement of virion attachment to target cells through direct interactions with bacterial molecules; modulation of host immunity by commensal bacteria promoting a tolerogenic environment permissive to viral replication; and enhancement of recombination potential through clustering of multiple virion particles for delivery during initial infection of host target cells (Figure 1). Current and future research on virus–bacteria interactions of various enteric pathogens, including emerging enteric viruses of interest (e.g., astroviruses), will likely reveal themes of shared mechanisms for bacterial enhancement of viral infection yet may also uncover novel mechanisms through which enteric viruses interact with the host microbiota.

One avenue of ongoing work aims to elucidate the effects of bacterial metabolites in shaping viral pathogenesis. Noroviruses benefit from association with bile acid compounds derived from bacterial metabolism. Another class of intestinal bacteria metabolites with potential roles in viral pathogenesis are the short chain fatty acids (SFCA) derived from the microbial breakdown of ingested carbohydrates in the intestinal lumen. It is reasonable to postulate that SFCA interact with enteric virus particles and impact pathogenesis similarly to bile acids. Additionally, both bile acids and SFCA are involved in complex physiological signaling processes spanning the digestive, nervous, endocrine, and immune systems. Research on the role of bacterial metabolites in the indirect shaping of viral pathogenesis through mediating host processes will lead to further insights on mechanisms of enteric virus pathogenesis and host responses to infection.
